# NASA Earth eXchange Hawaiʻi downscaled climate projections CMIP6 (NEX-HIDCP-CMIP6) dataset

**DOI:** 10.1016/j.dib.2026.113204

**Published:** 2026-08-27

**Authors:** Bridget Thrasher, Ian Brosnan, Abby G. Frazier, Kristen Sanfilippo, Matthew P. Lucas

**Affiliations:** a Climate Analytics Group LLC, Dayton, VA 22821, United States; b Earth Science Division, NASA Ames Research Center, Moffett Field, CA 94035, United States; c Graduate School of Geography, Clark University, Worcester, MA 01610, United States; d Department of Geography and Environment, University of Hawaiʻi at Mānoa, Honolulu, HI 96822, United States; e Water Resources Research Center, University of Hawaiʻi at Mānoa, Honolulu, HI 96822, United States

**Keywords:** Statistical downscaling, Climate change, Bias correction spatial disaggregation, BCSD

## Abstract

Hawaiʻi’s complex topography, pronounced microclimates, and geographic isolation make coarse-scale global climate model output inadequate for local planning, and high-resolution climate projections tailored to the state have until now been lacking. To address this gap, we introduce the NASA Earth eXchange Hawaiʻi Downscaled Climate Projections CMIP6 (NEX-HIDCP-CMIP6), a new archive of downscaled historical and future climate projections based on output from Phase 6 of the Coupled Model Intercomparison Project (CMIP6) over the State of Hawaiʻi. The dataset covers the years 1950–2100 and includes three variables (maximum temperature, minimum temperature, and precipitation) from five CMIP6 experiments (the historical experiment and the four Tier 1 scenarios: SSP1.2–6, SSP2.4–5, SSP3.7–0, and SSP5.8–5) as available from thirty CMIP6 models. The downscaled products are provided at 250-meter horizontal resolution and daily temporal resolution and were generated using the daily Bias Correction Spatial Disaggregation (BCSD) method against previously published 250-meter gridded observations from the Hawaiʻi Climate Data Portal. Technical validation confirms that the downscaled fields reproduce the expected geographic distribution of temperature and precipitation, with ensemble-mean precipitation generally within ±2% of the gridded observations and temperature means within 0.04 °C. NEX-HIDCP-CMIP6 complements two other NEX products, NEX-GDDP-CMIP6 and NEX-DCP30-CMIP6, fills a critical geographic gap in downscaled climate products for the United States, and is freely available through the NASA Advanced Supercomputing Data Portal.

Specifications Table

**Table:** 

Subject	Earth & Environmental Sciences
Specific subject area	Statistical downscaling of General Circulation Models (GCMs)
Type of data	Processed spatial data in NetCDF format
Data collection	CMIP6 output spanning 1950–2014 from the historical experiment and 2015–2100 from the future experiments (SSPs) were downloaded from the Earth System Grid Federation. Observational reference data for the period 1990–2014 were obtained from the Hawaiʻi Climate Data Portal.
Data source location	Data are stored at the NASA Advanced Supercomputing Data Portal.
Data accessibility	Repository name: NASA Advanced Supercomputing (NAS) Data Portal Data identification number: 10.25966/nex-hidcp-cmip6 Direct URL to data: https://data.nas.nasa.gov/nex-hidcp-cmip6
Related research article	

## 1 Value of the Data

• This dataset provides high-resolution, locally-specific climate information that is essential for effective planning and response in Hawaiʻi. By applying downscaling techniques, the latest global-scale climate projections have been translated into actionable information tailored to Hawaiʻi’s unique geography.
• This dataset supports a wide range of applications. Concrete examples of anticipated use include county water-supply agencies evaluating future groundwater recharge and reservoir inflows, agricultural extension services assessing shifts in growing-season temperature and rainfall, infrastructure planners siting roads and utilities exposed to changing extremes, conservation managers modeling shifts in species’ habitat ranges, and the insurance and finance sectors quantifying physical climate risk.
• Intercomparison studies between this dataset and earlier regional projections can help identify areas of agreement across models, methods, and scenarios and provide critical insight into the uncertainty and reliability of future rainfall projections.
• With four downscaled future scenarios derived from 30 GCMs, this dataset allows for evaluation and quantification of the uncertainty associated with both future emissions pathways and model structural differences.
• By explicitly incorporating Hawaiʻi into a CONUS-compatible framework, this dataset fills a critical geographic gap in downscaled climate products for the United States.

## 2 Background

The Hawaiian Islands’ geographic isolation and highly complex microclimates has challenged the development of regional climate projections for the state [1]. Although global-scale projections from numerical models have been made available in recent years through Phase 6 of the Coupled Model Intercomparison Project (CMIP6), the islands’ small size make those coarse-scale model outputs inadequate for local decision-making. Nevertheless, the recent release of both 250-m gridded observations over Hawaiʻi [2] and downscaled CMIP6 datasets from the NASA Earth eXchange (NEX-GDDP-CMIP6 [3] and NEX-DCP30-CMIP6 [4]) have provided the framework for producing a large ensemble of downscaled GCM projections for the state at high spatial resolution and daily temporal resolution. NEX-HIDCP-CMIP6 [5] is designed to facilitate direct comparisons between different scenarios and models, which will lead to a more comprehensive understanding of the variability and uncertainties inherent in these projections. Understanding climate projections for Hawaiʻi is crucial due to significant regional variability observed in previous research [6,7] and the severe impacts of future climate change [8]. This improved understanding is essential for building a more resilient Hawaiʻi.

Prior efforts to develop climate projections for Hawaiʻi have been constrained in resolution, temporal frequency, scenario coverage, or ensemble size. Dynamically downscaled projections based on CMIP5 [6] and elevation-dependent scaling approaches [7] advanced understanding of regional change but were limited to coarser spatial scales, a smaller set of models, and the earlier CMIP5 generation of scenarios. NEX-HIDCP-CMIP6 differs from and improves upon these earlier products in several respects: it is based on the more recent CMIP6 generation; it provides daily output for three variables across the full set of four Tier 1 SSP scenarios from thirty models; it is produced at 250-meter resolution consistent with the gridded observational record now available for the state [2]; and it is generated with the same daily BCSD framework used for the continental NEX-GDDP-CMIP6 [3] and NEX-DCP30-CMIP6 [4] archives, making it directly compatible with those CONUS-scale products. To our knowledge, no existing archive offers a daily, multi-model, multi-scenario downscaled CMIP6 ensemble for Hawaiʻi at this spatial resolution.

## 3 Data Description

The NEX-HIDCP-CMIP6 dataset is made available through the NASA Advanced Supercomputing (NAS) Data Portal. It consists of downscaled CMIP6 daily output of maximum temperature (tasmax, degrees C), minimum temperature (tasmin, degrees C), and precipitation (pr, mm/day) from the historical experiment (1950–2014) and four future scenarios (2015–2100; SSP1.2–6, SSP2.4–5, SSP3.7–0, and SSP5.8–5) as produced by thirty different climate models (Table 1). The spatial extent ranges from approximately 18.85–22.26 degrees North and 200.18–205.33 degrees East, at a horizontal resolution of 0.00225 degrees (approximately 250 m).

**Table 1:** CMIP6 models included in NEX-HIDCP-CMIP6 dataset. Notations refer to which of the four future scenarios (SSP1.2–6, SSP2.4–5, SSP3.7–0, and SSP5.8–5) are available from each model.

Model	pr	tasmax	tasmin
ACCESS-CM2	All	All	All
ACCESS-ESM1–5	All	All	All
BCC—CSM2-MR	All	All	All
CanESM5	All	All	All
CMCC-ESM2	All	All	All
CNRM-CM6–1	All	All	All
CNRM-ESM2–1	All	All	All
EC-Earth3	All	All	All
EC-Earth3-Veg-LR	All	All	All
FGOALS-g3	All	All	All
GFDL-CM4	2–4.5, 5–8.5	2–4.5, 5–8.5	2–4.5, 5–8.5
GFDL-ESM4	All	All	All
GISS-E2–1-G	All	All	All
HadGEM3-GC31-LL	1–2.6, 2–4.5, 5–8.5	1–2.6, 2–4.5, 5–8.5	1–2.6, 2–4.5, 5–8.5
HadGEM3-GC31-MM	1–2.6, 5–8.5	1–2.6, 5–8.5	1–2.6, 5–8.5
INM-CM4–8	All	All	All
INM-CM5–0	All	All	All
IPSL-CM6A-LR	All	All	All
KACE–1–0-G	All	All	All
KIOST-ESM	1–2.6, 2–4.5, 5–8.5	1–2.6, 2–4.5, 5–8.5	1–2.6, 2–4.5, 5–8.5
MIROC6	All	All	All
MIROC-ES2L	All	All	All
MPI-ESM1–2-HR	All	All	All
MPI-ESM1–2-LR	All	All	All
MRI-ESM2–0	All	All	All
NESM3	1–2.6, 2–4.5, 5–8.5	1–2.6, 2–4.5, 5–8.5	1–2.6, 2–4.5, 5–8.5
NorESM2-LM	All	All	All
NorESM2-MM	All	All	All
TaiESM1	All	All	All
UKESM1–0-LL	All	All	All

Each file in the dataset is in netCDF-4 classic format at deflation level 5 and contains one year of output, with the exact number of days per file determined by the calendar used by each model. The file sizes range from 265–285 megabytes (MB), and the total dataset volume is 9.2 terabytes (TB) (See Table 2 for a summary of the dataset attributes). The directory structure of the archive is as follows: https://data.nas.nasa.gov/nex-hidcp-cmip6/<model>/<experiment>/<realization>/<variable>. Similarly, the filenames are structured as follows: <*variable*>_day_<*model*>_<*experiment*>_<*realization*>_<*grid*>_<*year*>.nc. For example, the file pr_day_ACCESS-CM2_historical_r1i1p1f1_gn_1973.nc is located in the directory https://data.nas.nasa.gov/nex-hidcp-cmip6/ACCESS-CM2/historical/r1i1p1f1/pr.

**Table 2:** Summary of the NEX-HIDCP-CMIP6 dataset attributes.

Attribute	Description
Variables	Maximum temperature (tasmax), minimum temperature (tasmin), and precipitation (pr)
Units	Degrees Celsius (tasmax, tasmin); millimeters per day (pr)
Temporal coverage	1950–2014 (historical); 2015–2100 (SSP scenarios)
Temporal frequency	Daily
Experiments / scenarios	Historical, SSP1.2–6, SSP2.4–5, SSP3.7–0, and SSP5.8–5
Models	Thirty CMIP6 models (see Table 1 for per-model scenario availability)
Spatial resolution	0.00225 degrees (approximately 250 m)
Spatial extent	Approximately 18.85–22.26° N, 200.18–205.33° E
File format	netCDF-4 classic, deflation level 5; one year per file
File size	265–285 MB per file
Total volume	9.2 TB
Repository	NASA Advanced Supercomputing (NAS) Data Portal
DOI	10.25966/nex-hidcp-cmip6
Data URL	https://data.nas.nasa.gov/nex-hidcp-cmip6
Downscaling code	https://doi.org/10.5281/zenodo.18525436

## 4 Experimental Design, Materials and Methods

**Fig. 1:**
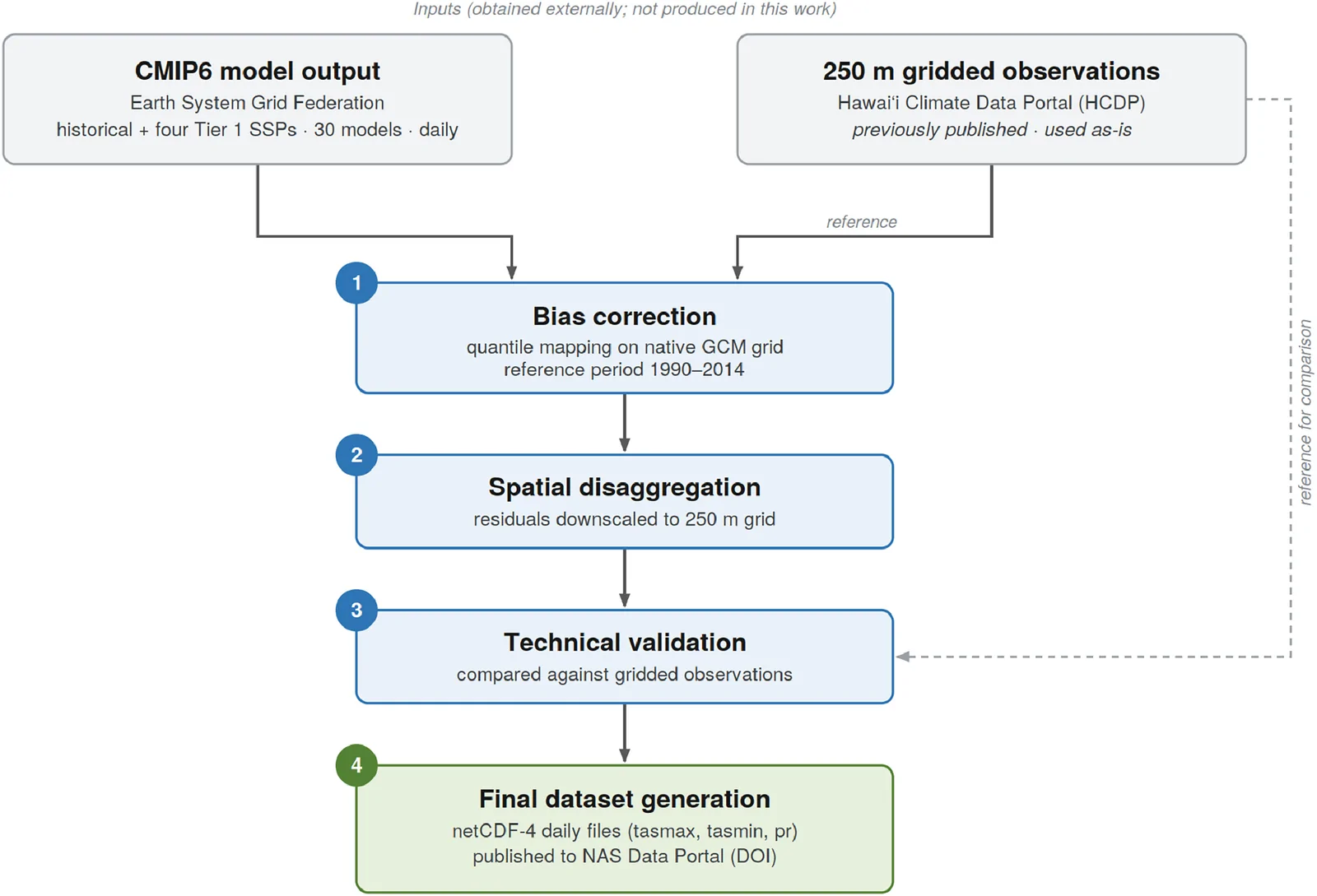
Workflow for generating the NEX-HIDCP-CMIP6 dataset. CMIP6 model output and the 250-meter gridded observational dataset serve as inputs. The processing performed comprises bias correction of the model output against the observational reference, spatial disaggregation to the 250-meter grid, technical validation against the observations, and final dataset generation.

NEX-HIDCP-CMIP6 was produced by applying the daily BCSD method to CMIP6 model output, using an existing high-resolution observational dataset as the downscaling reference. Two inputs were required. Daily output from thirty CMIP6 models was retrieved from the Earth System Grid Federation for the historical experiment and the four Tier 1 SSP scenarios. The 250-meter daily gridded observations of rainfall and temperature for Hawaiʻi, a previously published product [2], were obtained from the Hawaiʻi Climate Data Portal and used as-is as the reference dataset. The downscaling then proceeded in three steps: bias correction of the model output against the observational reference, spatial disaggregation to the 250-meter grid, and technical validation of the downscaled products against the gridded observations. The overall workflow is summarized in Fig. 1, and each input and step is described in detail below.

### 4.1 Daily gridded observations

The 250-meter gridded observations used as the downscaling reference are an existing, previously published product and were not produced as part of this work. The methodology summarized in this section is that of the cited sources and is provided here for context. Daily gridded historical rainfall and temperature data from 1990 to 2014 were obtained from the Hawaiʻi Climate Data Portal (HCDP; https://www.hawaii.edu/climate-data-portal/) [2]. The HCDP collects observation data from multiple sources, including the Hydrometeorological Automated Data System (HADS), the National Center for Environmental Information (NCEI), the Soil Climate Analysis Network (SCAN), various local networks and repositories (including the Hawaiʻi Mesonet), and a previously published dataset covering the period 1990–2014 [9]. These data are converted into a consistent standard format, including SI units, time stamps, and time steps. The data then undergo manual quality control, screening for outliers, and are then aggregated to daily 24-hour accumulated values for rainfall.

Gaps in the daily rainfall record were partially filled using the normal ratio method [10]. To produce daily rainfall grids at 250 m spatial resolution, log-transformed relative daily rainfall anomalies were calculated by dividing daily observations by monthly means [11], then testing fixed or free fitting variograms for seven methods with different regularization terms. R's autoKrige function [17] then generated 14 potential rainfall maps per day by applying both daily-fitted and fixed monthly variograms to each method. Finally, leave-one-out cross-validation identified the daily map with the lowest RMSE, a process that improved accuracy over a previous study [9]. For temperature, the network of stations is much smaller, and daily maps were produced using an optimized piecewise linear regression technique. (See Kodama, et al., [12] for additional details.)

### 4.2 CMIP6 global climate model outputs

Outputs from the Coupled Model Intercomparison Project Phase 6 (CMIP6) are available through the Earth System Grid Federation [13]. The climate models included in NEX-HIDCP-CMIP6 are the same as those included in the other NEX downscaled CMIP6 archives (NEX-GDDP-CMIP6, NEX-DCP30-CMIP6) and were chosen based on whether their output included both the historical and at least two SSP experiments at the time of data retrieval. For those models that produced multiple realizations of each experiment, only a single variant was downscaled. Because identical variant labels did not signify identical configurations across models in CMIP6, each variant was chosen to maximize the level of completeness across SSPs and variables as available from each model.

### 4.3 Daily BCSD statistical downscaling method

**Fig. 2:**
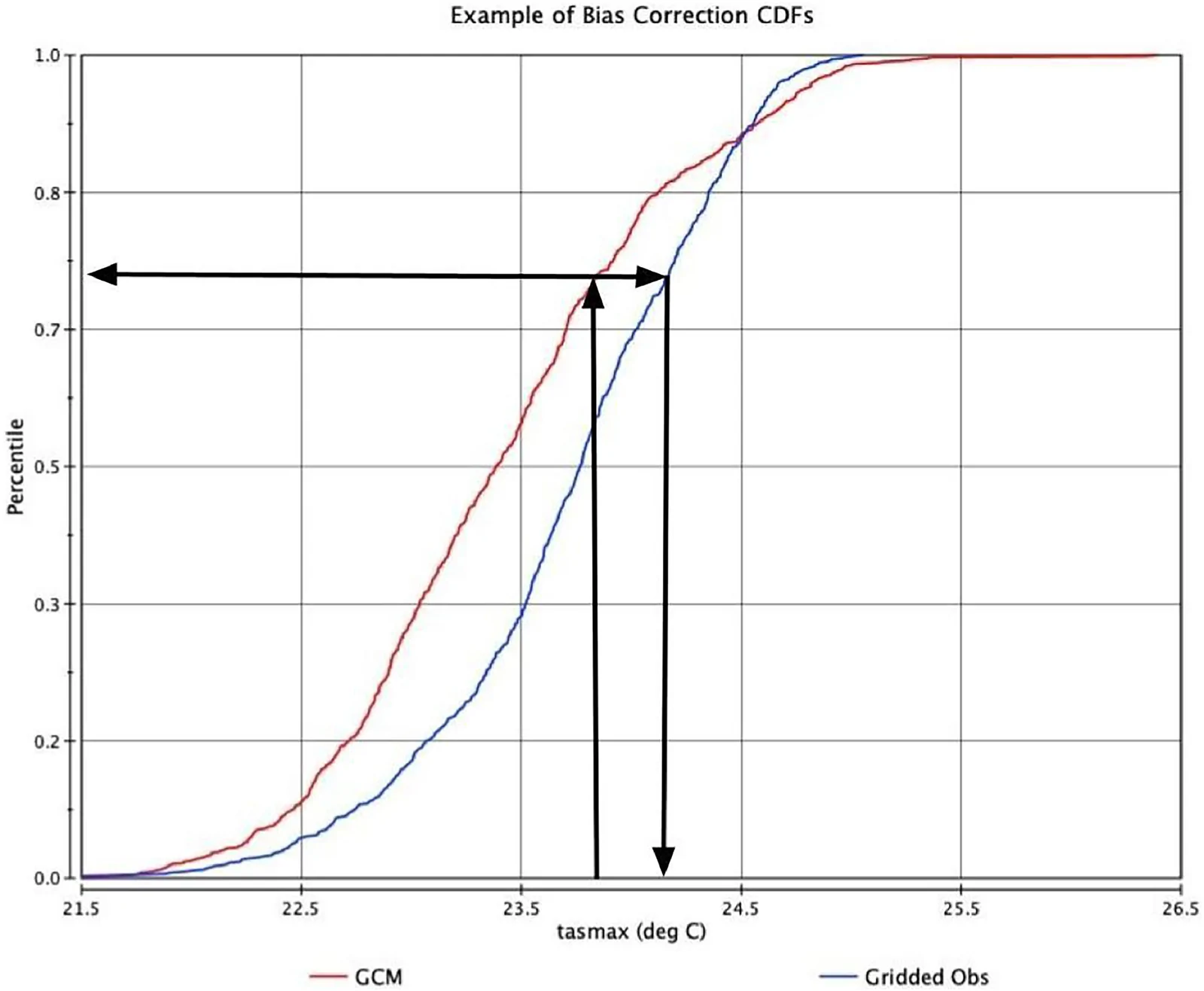
Example of the bias correction process.

The daily Bias Correction Spatial Disaggregation (BCSD) statistical downscaling method [14] was applied as described in Thrasher et al., 2022 [15] and 2024 [16]. The bias correction was implemented on the native GCM grid using quantile mapping (Fig. 2) with a ± 15-day moving window over the reference period 1990–2014. This resulted in 775 values forming each cumulative distribution function. (See Fig. 2.) The spatial disaggregation was applied to the residuals after removing the GCM-scale daily climatology, after which the observation-scale daily climatology was restored.

**Fig. 3:**
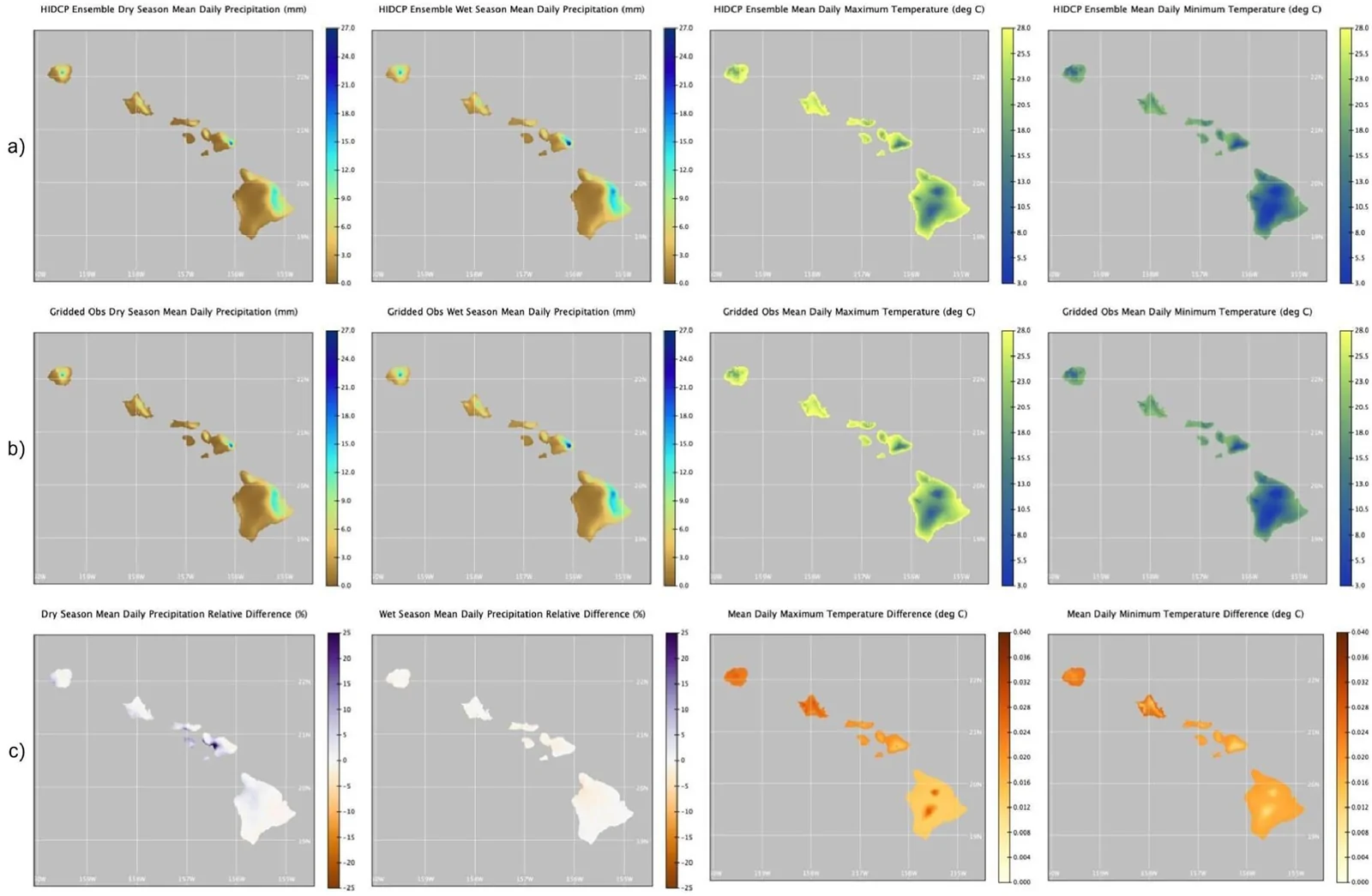
Mean daily values of dry season (May-October) precipitation, wet season (November-April) precipitation, maximum temperature, and minimum temperature for 1990–2014. Row a) shows the HIDCP ensemble means, row b) shows the gridded observation means, and row c) shows the differences.

**Fig. 4:**
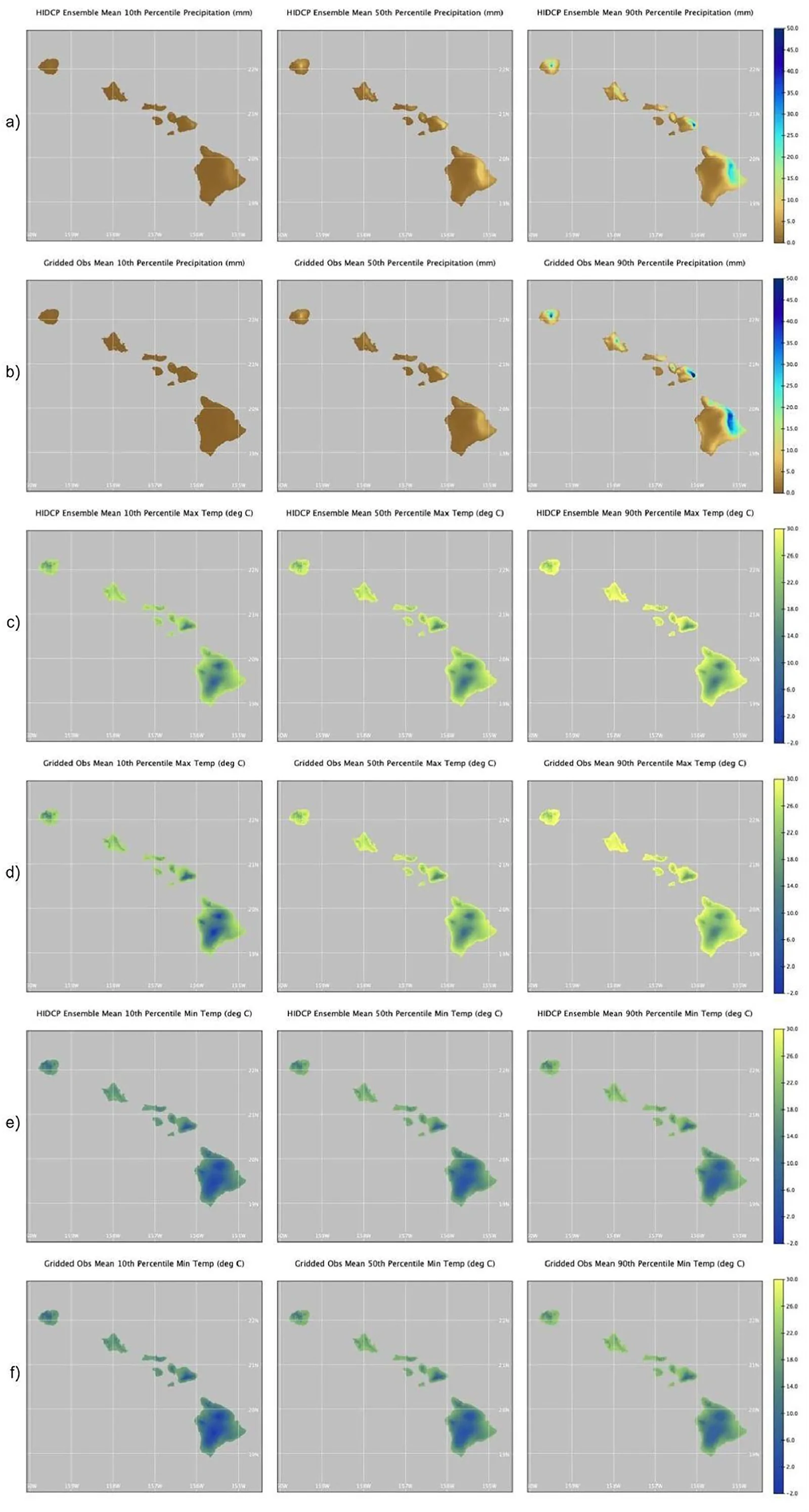
The mean 10th, 50th, and 90th percentiles: a) HIDCP ensemble mean daily precipitation, b) obs mean daily precipitation, c) HIDCP ensemble mean daily maximum temperature, d) obs mean daily maximum temperature, e) HIDCP ensemble mean daily minimum temperature, and f) obs mean daily minimum temperature.

After the downscaling processing was complete, a technical validation was conducted to confirm the method performed as expected. Fig. 3 compares the mean daily values over the reference period for dry season (May-October) precipitation, wet season (November-April) precipitation, maximum temperature, and minimum temperature from both the HIDCP ensemble and the gridded observations. The geographical distribution of the downscaled values are as expected (e.g., highest precipitation occurs on the windward side, lowest temperatures occur at higher elevations). Most precipitation values differ within ± 2%, with larger deviations present where slight differences in trace rainfall amounts can lead to large percentage differences. In both maximum and minimum temperatures, the HIDCP ensemble means are larger than the gridded observation means by up to 0.04 degrees Celsius. Fig. 4 illustrates the median values as well as extremes for each variable (10th, 50th, and 90th percentiles). As anticipated, the percentile values exhibit a geographically consistent distribution. Users are responsible for any further validation of these data as required for specific applications.

### 4.4 Downscaling code

The NCL code used to generate the downscaled products can be found at 10.5281/zenodo.18525436. The downscaling was implemented in NCL (version 6.6.2) and executed on the NASA Advanced Supercomputing facility supercomputer. The 250-meter gridded observations used as the reference are a previously published product [2], so the software and interpolation methods used to produce them are described in the cited sources rather than repeated here.

## Limitations

The high spatial and temporal resolution of this dataset can require significant computational resources for storage, processing, and analysis. In addition, the daily BCSD method assumes that relative spatial patterns in future climate change will remain consistent with those represented in the gridded observation reference dataset. Lastly, the downscaled values corresponding to extreme events in the GCM output might be underestimated or overestimated in some locations due to dispersal of the area-averaged value that results from the interpolation from low to high spatial resolution. However, the frequency of those extreme events within each individual scenario are preserved.

Several additional sources of uncertainty should be noted. First, the spread across the thirty CMIP6 models reflects structural differences in model formulation. No single model or ensemble member should be treated as a definitive projection, and users are encouraged to consider the full multi-model range when characterizing uncertainty. Second, the downscaled products inherit any biases and limitations of the gridded observational reference dataset, including reduced station density in some regions and at higher elevations, which can affect the quality of the reference fields used for bias correction. Third, although the products are provided at 250-meter resolution, they should be applied with caution at very local scales, where fine-scale processes not represented in either the observational reference or the downscaling method may be important.

## Ethics Statement

No human subjects, animal experiments, or social media data were used in the creation of the NEX-HIDCP-CMIP6 dataset. All authors have read and followed the ethical requirements for publication in Data in Brief.

## CRediT Author Statement

**Bridget Thrasher:** Conceptualization, Methodology, Software, Data Curation, Writing - Original Draft.

**Ian Brosnan:** Conceptualization, Resources, Writing - Review & Editing, Funding acquisition.

**Abby G. Frazier:** Conceptualization, Writing - Original Draft.

**Kristen Sanfilippo:** Writing - Original Draft.

**Matthew P. Lucas:** Conceptualization, Resources.

## Data Availability

NASA Advanced Supercomputing Data PortalNEX-HIDCP-CMIP6 (Original data)
